# Artificial intelligence platform to predict children's hospital care for respiratory disease using clinical, pollution, and climatic factors

**DOI:** 10.7189/jogh.15.04207

**Published:** 2025-07-21

**Authors:** William Cabral-Miranda, Cauê Beloni, Felipe Lora, Rogério Afonso, Thales Araújo, Fátima Fernandes

**Affiliations:** 1 Instituto de Pesquisa e Ensino em Saúde Infantil (PENSI Institute) – José Luiz Setúbal Foundation, São Paulo, Brazil; 2 Sabará Children’s Hospital, São Paulo, Brazil

## Abstract

**Background:**

Hospitals and health care systems may benefit from artificial intelligence (AI) and big data to analyse clinical information combined with external sources. Machine learning, a subset of AI, uses algorithms trained on data to generate predictive models. Air pollution is a known risk factor for various health outcomes, with children being a particularly vulnerable group.

**Methods:**

This study developed and validated an AI-based platform to forecast paediatric emergency visits and hospital admissions for respiratory diseases, using clinical and environmental data in the Metropolitan Area of São Paulo, Brazil. We applied XGBoost, a tree-based machine learning algorithm, to predict hospital use at Sabará Children’s Hospital, incorporating clinical, pollution, and climatic variables.

**Results:**

We analysed 24 366 emergency department visits and 2973 hospital admissions for respiratory diseases International Classification of Diseases, 10th Revision, Chapter J (ICD-10 J), excluding COVID-19, from January to December 2022. Only geocoded cases within the spatial accuracy thresholds of the study were included. Logistic regression revealed that outpatient visits were associated with higher particulate matter with a diameter of 10 µm or less (PM_10_) concentrations near children’s residences on the day of hospital arrival. In contrast, admissions were linked to lower relative humidity, particularly on drier days. Additional associations were found between admissions and the spring season, as well as male sex.

**Conclusions:**

We developed a platform that integrates clinical and environmental databases within a big data framework to process and analyse information using AI techniques. This tool predicts daily emergency department and hospital admission flows related to paediatric respiratory diseases. The algorithms can distinguish whether a child arriving at the emergency department is likely to be treated and discharged or will require hospital admission. This predictive capability may support hospital planning and resource allocation, particularly in contexts of environmental vulnerability.

Artificial Intelligence (AI) and Big Data offer new possibilities for health care systems to analyse clinical data by integrating them with external sources such as environmental and demographic data sets [1,2]. Within this field, Machine Learning (ML) algorithms can detect patterns in complex data sets and generate predictive models to support real-time clinical decision-making [2–4].

Recent advances in ML have shown great promise in health care, particularly in predicting clinical outcomes such as morbidity, hospital readmission, and mortality [5,6]. These models can also support hospital management by forecasting patient flows, which is crucial in high-demand settings like emergency departments. For instance, Assistance Publique-Hôpitaux de Paris has tested predictive systems based on hospital admissions, meteorological data, and influenza incidence patterns [1].

In parallel, environmental factors – especially air pollution – have been consistently associated with increased risks of respiratory diseases, particularly among children, who represent a highly vulnerable group [7–9]. Studies conducted in urban centres have demonstrated strong associations between daily variations in air pollution and paediatric emergency visits for respiratory conditions [10]. In the São Paulo Metropolitan Area, for instance, over 70% of the population is exposed to ozone (O_3_) levels above World Health Organization (WHO) guidelines, with current state-level alert thresholds exceeding WHO recommendations [11,12].

Hospital Infantil Sabará, a major paediatric hospital in São Paulo, reports that approximately 60% of its emergency department cases are related to respiratory diseases. This presents both a challenge and an opportunity: the possibility of developing predictive tools that can help anticipate peaks in demand, especially when environmental conditions are likely to exacerbate respiratory conditions.

This study aims to develop and validate a predictive platform based on AI that integrates clinical and environmental data to forecast paediatric emergency visits and hospital admissions for respiratory diseases in the São Paulo Metropolitan Area. The central research question is: to what extent can AI techniques, integrating clinical and environmental data, accurately forecast paediatric emergency visits and hospital admissions for respiratory illnesses in an urban setting?

## METHODS

### Data sources

#### Study design and location

This is a hospital-based study utilising data from emergency department visits and hospital admissions at Sabará Children's Hospital, where cases will be analysed with data of air pollution and urban characteristics. The study purpose to explore and generate hypotheses for future investigations, as well as to test pre-established hypotheses regarding health conditions, environmental factors, urban features and clinics aspects.

This research utilises the XGBoost an AI-based ML model to predict respiratory disease in a children's hospital in São Paulo, Brazil incorporating clinic and climatic factors. All methodology stages are described the following topics.

The Python software, version 3.10 (Python Software Foundation, Wilmington, DE, USA) was chosen due to its robust capabilities in statistical computation and graphics visualisation as well as the capacity for the development of computer systems. This research in addition to proposing a predictive statistical analysis based on ML, we developed a computational platform with AI and Big Data foundations to process, manage, visualise and analyse large clinical databases and external data.

### Data collection

The clinical database consists of the following variables: the code for the service generated on the day of the appointment, the date of attendance in the emergency department, the type of service (outpatient, hospital admissions, emergency), the hospitalisation bed, the expected discharge date, the actual discharge date, the International Classification of Diseases (ICD) code, gender, and age.

Residential information includes street, house number, postal code, city, and state.

The pollution and climate variables include daily averages of particulate matter (PM_10_), O_3_, temperature, and humidity [13].

### Preprocessing

#### Geocoding patient addresses

We used geocoding of patients' addresses to generate pairs of geographic coordinates, latitude and longitude, to extract information about the surroundings of the homes of patients involved in the research.

To obtain geolocation, the Opencage Data Api was used. However, it is not always possible to determine locations with high precision for certain addresses. To identify such scenarios, the confidence field from the Opencage Data Api response was utilised, which indicates the degree of confidence in the geolocation accuracy (**Table 1**).

**Table 1:** Geocoding confidence levels

Confidence	Meaning/distance (in km)
10	<0.25
9	<0.5
8	<1
7	<5
6	<7.5
5	<10
4	<15
3	<20
2	<25
1	>25
0	unable to determine a bounding box, thus unable to determine a confidence

All geolocations with a confidence level below eight were excluded from the model's training. The main reasons for the inability to determine geolocation with high precision include incorrectly entered or incomplete address information.

#### Interpolation of air pollution and atmospheric conditions index

The atmospheric condition data – namely inhalable particles (PM_10_), ozone (O_3_), temperature, and air humidity – were obtained from Environmental Company of the State of São Paulo stations located at various points across the Greater São Paulo region.

Specifically, PM_10_ concentrations ranged from 0–250 μg/m^3^, O_3_ from 0–200 μg/m^3^, temperature from 0–40°C, and air humidity from 0–100%. These variables were selected to represent key atmospheric conditions potentially related to paediatric respiratory health outcomes.

To infer air quality across the entire region, the Inverse Distance Weighting method provided by the Pyinterpolate library was used.

#### Oversampling strategy

To address class imbalance in the training data set we applied the Synthetic Minority Over-sampling Technique. This technique generates synthetic examples of the minority class based on feature space similarities between existing minority instances. The Synthetic Minority Over-Sampling Technique algorithm was implemented using the ‘imblearn’ Python library and was applied exclusively to the training set to prevent data leakage [14].

### Platform development

We internally defined a module with business rules and database communication, responsible for managing platform users and interfacing with data sources. This application integrates messaging (RabbitMQ), a database (MySQL), and the ML module.

We developed modules for user management, a dashboard for real-time data visualisation, charts for displaying variable information, tables for viewing patient data with filters and export capabilities, a map visualisation with patient residence locations, and levels of climatic and pollution conditions. These modules may handle asynchronous processes or those requiring longer processing times.

All applications were developed using Docker containers and deployed with the Kubernetes container orchestrator in a two-node cluster. This setup allows easy scaling of any module to accommodate increased access volumes or processing demands. Each node features a server with two cores and four gigabytes of memory, added to the cluster with 100 gigabytes of storage.

### Model development

#### Model training

We trained the XGBoost model using the features and fine-tuned hyperparameters. The training procedure entails dividing the data set into separate training and validation sets (75 and 25%, respectively), we adjusted the model parameters to minimi**s**e the loss function. The training **–** validation split partitions the data set into separate training and validation sets to assess the performance of the model on unknown data [15].

The selection of XGBoost is based on its robustness, efficiency, and capacity to effectively process extensive data sets with intricate interactions. Regulari**s**ation techniques were employed to avoid overfitting [15].

### Statistical analysis

The risk analysis for hospital admission was conducted using the XGBoost ML model, which demonstrated the best performance across evaluation metrics. Additionally, decision tree and logistic regression models were tested and compared to assess predictive capabilities. These ML approaches were primarily employed to support the development of alert systems, given their ability to handle complex interactions and generate accurate, real-time risk forecasts.

For forecasting the number of consultations and hospital admissions over time, a temporal logistic regression model was employed. Furthermore, a conventional logistic regression analysis was carried out to examine whether specific environmental and clinical variables were associated with emergency department visits and hospital admissions, independently. While ML models are advantageous for prediction and real-time alerting in public health contexts, traditional regression remains particularly suitable for understanding the epidemiological relationships between environmental exposures and health outcomes.

### Evaluation metrics

#### Assessment

The evaluation of the XGBoost model assessed its performance using metrics including accuracy, sensitivity (recall), specificity, area under the curve (AUC), and the receiver operating characteristic curve (ROC), cross-validation (10-fold) and standard deviation [16].

## RESULTS

Sabará Children’s Hospital provides care to approximately 100 000 paediatric patients annually, predominantly from the state of São Paulo and various regions across Brazil. Of these, nearly 50% present with respiratory conditions. For this study, we included only cases meeting the following criteria:

1. a confirmed diagnosis of respiratory disease recorded in the hospital’s clinical information system

2. successful geocoding of the residential address with high spatial accuracy – levels 8–10 on the geolocation confidence scale (**Table 1**)

3. residence within the São Paulo Metropolitan Area.

We analysed 24 366 emergency department visits and 2973 hospital admissions due to respiratory diseases (ICD-10 J, excluding COVID-19 occurring between January–December 2022.

In this data set, the weighted average patient age was 3.9 years, with 54.22% of cases involving male children. Respiratory diseases account for a significant proportion of patient flow at the hospital, representing approximately 60% of all general visits.

We employed the XGBoost, decision tree and logistic regression ML algorithm to predict whether cases presenting with respiratory diseases at the emergency department would result in outpatient treatment or hospital admission. Prediction performance was evaluated using accuracy, sensitivity, specificity, error rate, cross-validation, standard deviation and ROC curve metrics.

After oversampling, performance metrics showed substantial improvements, with the model demonstrating reliable predictive capacity using the selected algorithm and input variables.

Among the ML models evaluated, the XGBoost classifier demonstrated the most favourable performance. It achieved the highest overall accuracy and the lowest error rate, while maintaining a strong balance between sensitivity and specificity. The model also exhibited the highest area under the ROC curve, indicating superior discriminative ability compared to logistic regression and decision tree. Despite the decision tree model reaching the highest sensitivity, it showed lower specificity and higher error, which compromises its reliability in distinguishing negative cases. The XGBoost model also showed robust performance in cross-validation, suggesting good generalisation across data folds (**Table 2**).

**Table 2:** Result parameters of predictive machine learning models using oversample

Model	Accuracy (%)	Sensitivity (recall) (%)	Specificity (%)	Error (%)	Cross-validation	standard deviation	ROC curve
XGBoost	85	83	89	15	0.88	0.006	0.81
Decision tree	73	87	71	27	0.88	0.010	0.79
Logistic regression	81	78	81	19	0.89	0.006	0.80

ROC – receiver operating characteristic

**Figure 1:**
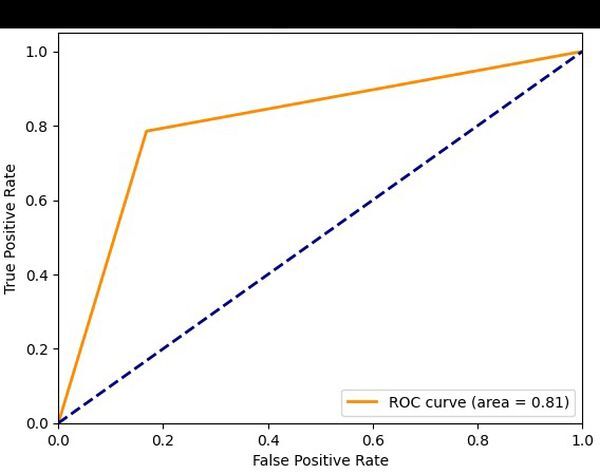
Result of receiver operating characteristic curve (ROC) and area under the curve (AUC) curve.

To further evaluate model performance across different classification thresholds, we used the ROC curve. The model achieved an AUC value of 0.81, indicating strong discrimination between classes (**Figure 1**). An AUC closer to one suggests excellent model performance, while a value near 0.5 indicates no better than random chance.

For aggregated daily predictions of the number of hospital visits and admissions, we applied temporal logistic regression models using the scikit-learn library, structured to incorporate temporal predictors. These models estimate the probability of hospital utilisation at the population level as a function of daily environmental exposures (*e.g*. air pollution, temperature, humidity) and temporal patterns (*e.g*. day of the week, seasonal trends). This approach was selected for its interpretability, ease of implementation, and suitability for integration into a public health surveillance framework.

**Figure 2:**
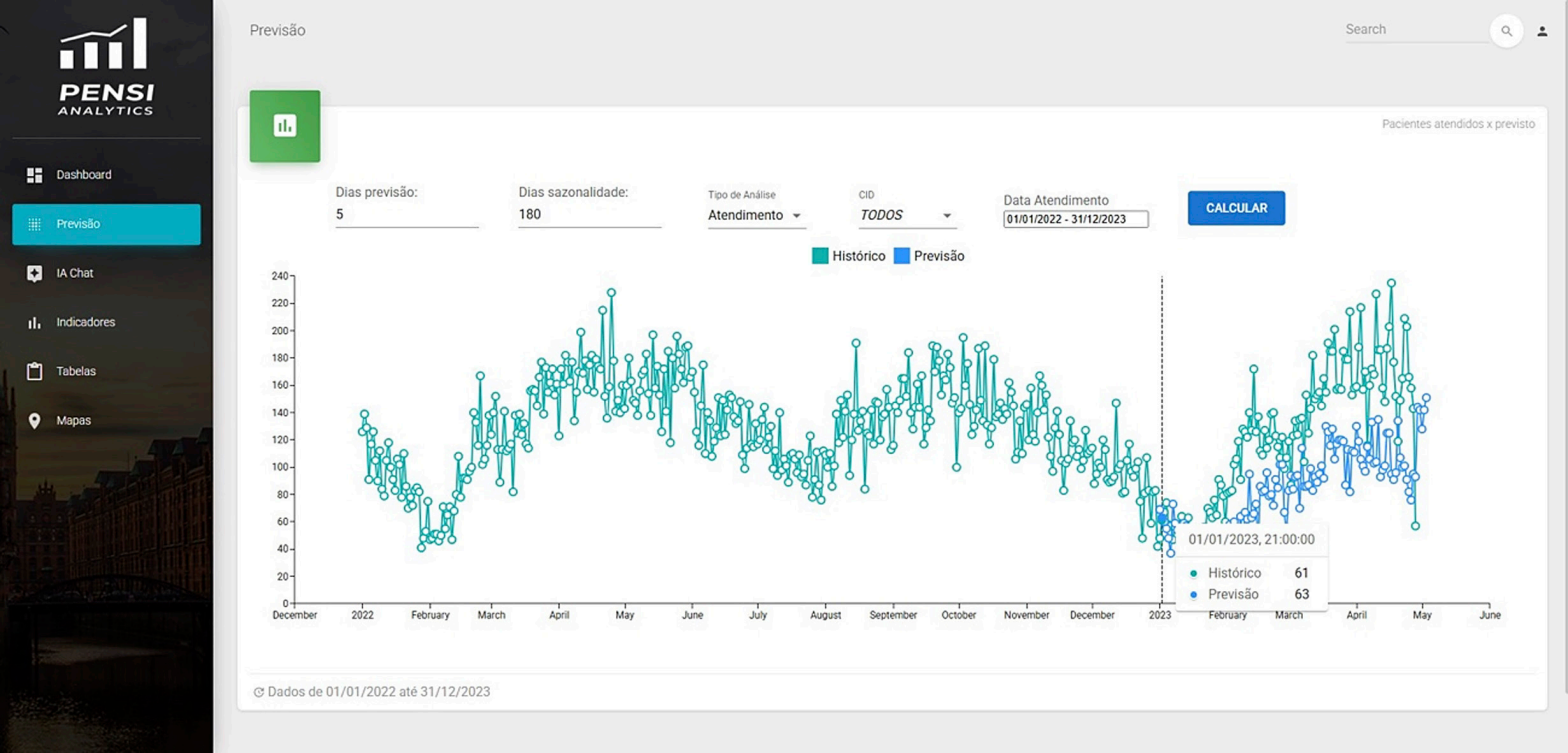
Visualisation of the daily hospital forecast graph.

The models were trained to forecast daily case counts for both emergency department visits and hospital admissions, stratified by ICD codes, with prediction horizons of five, 10, and 30 days in advance. This forecasting capacity enables early anticipation of demand, which is particularly relevant for operational planning and resource allocation in paediatric hospitals. **Figure 2** illustrates the model's predicted volumes of respiratory-related visits in the days following the analysis period.

For instance, on 1 January 2023, the model predicted 63 outpatient cases, closely matching the reported 61 (**Figure 2**). On 2 February, it predicted 86 cases *vs*. 102 reported; 3 February: 108 predicted, 105 reported; 4 February: 117 predicted, 118 reported; 5 February: 98 predicted, 147 reported; 6 February: 101 predicted, 112 reported; and 7 February: 154 predicted, 107 reported. The model achieved an average error of 18 cases over this seven-day forecast period.

Using logistic regression, we identified that emergency department visits due to respiratory conditions were associated with elevated PM_10_ concentrations near patients’ residences on the same day they sought care in the emergency department (**Table 3**).

**Table 3:** Results of logistic regression analyses for attendances and hospital admissions as dependent variables

Attendances as the dependent variable				95% CI	
Variable	Beta	*P*-value	Beta (exp)	Lower	Upper
PM_10_	0.06	0.002	1.006	1.002	1.010
**Hospital admissions as the dependent variable**				**95% CI**	
Variable	Beta	*P*-value	Beta (exp)	Lower	Upper
Relative air humidity	−0.07	0.01	0.999	0.987	0.999
Male	0.12	0.002	1.128	1.045	1.217
Spring	0.31	0.0001	1.368	1.206	1.552

CI – confidence interval, PM_10_ – particulate matter

Hospital admissions, in contrast, were more likely to occur on drier days, characterised by lower relative humidity in the areas where patients resided. Additionally, we found hospital admissions to be associated with the spring season and a higher prevalence among male patients (**Table 3**).

In this study, predictive modelling and association analysis were performed using two complementary methods. The XGBoost algorithm was implemented to develop an early warning system capable of predicting paediatric emergency department visits and hospital admissions and due to respiratory diseases, based on atmospheric pollution levels and urban characteristics. XGBoost was selected for its ability to capture complex, nonlinear interactions among variables, handle large and high-dimensional data sets, and optimise predictive performance, which are essential features for practical applications in health surveillance. Simultaneously, conventional logistic regression was applied to assess and quantify the independent associations between predictors and health outcomes. The logistic regression model allowed for direct estimation of effect sizes and provided interpretable, statistically validated insights into the relative contribution of each factor, thus complementing the predictive findings and enhancing the epidemiological understanding necessary for evidence-based decision-making.

We developed a platform capable of integrating clinical databases with external data within a Big Data framework, designed to process and analyse information using AI techniques applied to health care – particularly paediatric health, due to its unique characteristics and conditions compared to the adult population.

**Figure 3:**
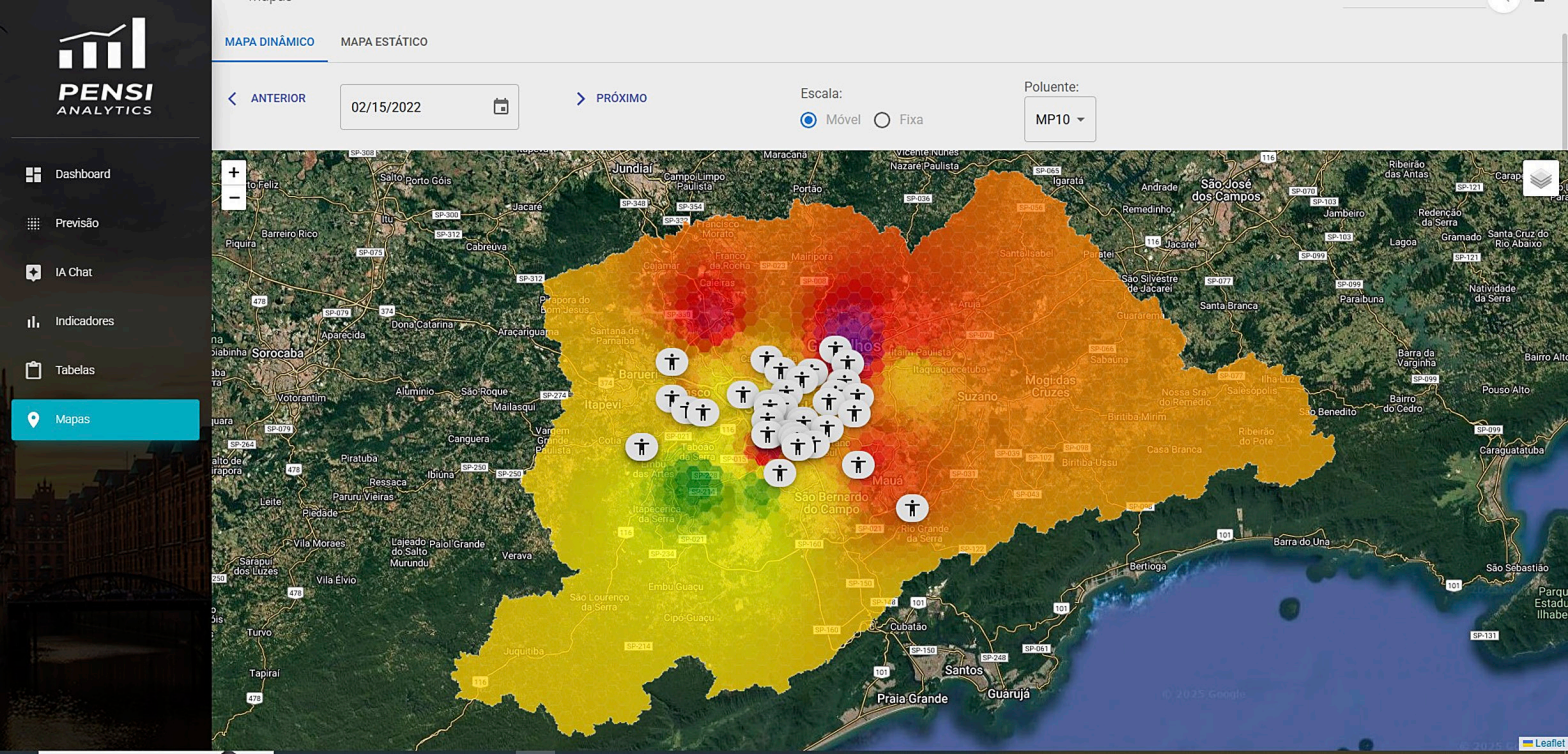
Spatial components visualisation interface for integration with external and clinical data.

A central feature of this platform is the automation of all research-related processes, resulting in increased efficiency and precision in analysing large volumes of data from diverse sources. This includes the retrieval of data from hospital management systems containing clinical records and patient information, followed by the geocoding of residential addresses (**Figure 3**).

Data acquisition related to pollution levels and atmospheric conditions is also automated through public data platforms connected to environmental monitoring stations. Interpolation techniques are applied to generate spatial layers of environmental data, allowing for the estimation of daily exposure levels for individual patients.

Subsequently, predictive analyses were carried out using ML and logistic regression models to forecast emergency department visits and hospital admissions at a paediatric hospital.

## DISCUSSION

In Wuhan, China, seasonal fluctuations in daily hospital admissions counts were observed, with higher rates occurring in spring and winter compared to summer and autumn [17,18]. Each 10 μg/m^3^ increase in PM_10_ concentration was associated with a rise in hospital admissions due to respiratory diseases. Age-specific analyses revealed that this effect was more pronounced in the 0–14-year age group [18]. Similarly, an ecological study conducted in Argentina demonstrated a significant association between elevated PM_10_ levels and an increase in medical consultations on the fourth day following exposure [19].

An evaluation of hospital admissions risks for respiratory diseases among children in São Paulo's administrative districts found that PM_10_ concentrations exceeding 35 μg/m^3^ were significantly associated with increased cumulative relative risk (RR) – particularly among female children – beginning from the day of exposure. In the general population, a significant risk increase was only observed on the day following exposure. The highest RR was recorded at lag 1, reaching 1.512 (95% confidence interval = 1.914–2.067) for female patients at high exposure levels. Conversely, lower concentrations of PM_10_ were associated with reduced hospital admissions risks [20].

Gu et al. (2024), in a literature review, reported that asthma patients over 14 years of age exhibited a higher RR of seeking medical care when exposed to 95% relative humidity, compared to lower humidity levels [21]. Additionally, studies have shown that low humidity can increase the concentration of particulate matter, thereby elevating the risk of respiratory diseases.

In Suzhou, China, an analysis of dominant respiratory pathogen detection rates revealed a negative correlation with average monthly precipitation, but no significant association with monthly relative humidity. Positive correlations were observed with PM_10_ concentrations, while negative correlations emerged with average monthly temperature, wind speed, and O_3_ levels. A positive correlation was also found with carbon monoxide concentrations [17].

Several ecological studies have identified statistical associations between higher relative humidity and increased cases of respiratory diseases [20,22,23]. However, Amorim et al. (2013) demonstrated that drier days were linked to bronchitis cases in Manaus, a city in the Amazon region [23]. In our study, we similarly found that hospital admissions for respiratory illnesses were more frequent on days with lower relative humidity in residential areas surrounding Sabará Children’s Hospital.

Information on the relationship between climate change and population health has largely been generated in high-income countries. This imbalance creates critical knowledge gaps in low- and middle-income countries, where limited scientific evidence may hinder informed decision-making and compromise effective climate change mitigation policies [24].

The consequences of these challenges are evident in increasingly erratic weather patterns. The task of fostering healthier environments has become one of the most pressing global issues of our time [25]. Addressing climate and child health concerns requires a systemic approach that capitalises on ongoing climate initiatives. When research, policy, and action are synergistically aligned, countries can improve public health, enhance air quality, and create healthier urban environments [26].

Such a perspective is essential for shaping effective public health and climate policies and raising awareness about the urgent need for action [26]. For example, the project ‘Increasing the Health Sector’s Capacities and Strengthening Coordination on Climate Action in Argentina at National and Subnational Levels’ exemplifies early efforts to integrate research and policy by engaging diverse stakeholders – including government, academia, the private sector, and local communities.

The findings of this study have significant implications for public health planning and policy in São Paulo. The predictive model, by accurately identifying periods and environmental conditions associated with increased risk of respiratory illnesses in children, can serve as a foundation for developing early warning systems that alert health care providers and the public about impending high-risk days. Such systems would allow hospitals to optimise resource allocation, including staff deployment and bed availability, thereby improving readiness and response to surges in paediatric admissions. Moreover, integrating these predictive insights into local environmental health policies could support more proactive regulatory actions on air pollution control. By bridging clinical forecasting and environmental monitoring, this approach promotes a comprehensive strategy for mitigating the health impacts of urban air pollution and enhancing resilience within the health care system.

Effective health impact planning requires measurable indicators, active engagement across sectors, and transparent funding mechanisms. This can be achieved through the development of comprehensive, intersectoral, and transdisciplinary databases and observatories. These resources can bridge health and other data, facilitating the development of health systems [26].

Collectively, these studies support the integration of climatic variables into predictive models as a means to enhance public health surveillance. The high predictive accuracy of the XGBoost model in our study underscores the potential of ML to transform public health strategy and strengthen global disease prevention and control efforts [27].

The performance of the XGBoost model, reflected by an AUC of 0.81 on the ROC curve, demonstrates a strong discriminatory capacity for predicting emergency department visits and hospital admissions due to respiratory diseases in children. Practically, this indicates that the model is effective in accurately identifying children at higher risk of developing severe clinical conditions, based on both environmental (air pollution and climatic variables) and clinical data. This finding is particularly relevant in the urban context of São Paulo, where fluctuations in pollutants such as PM_10_ and O_3_ are common.

The integration of environmental variables into the predictive framework enables the early anticipation of respiratory disease outbreaks linked to poor air quality, thereby supporting preventive actions within the health care system. These may include increasing hospital capacity on critical days, issuing public health alerts, and strategically allocating health care resources. Therefore, these findings not only represent a methodological advance in the use of AI in health forecasting, but also offer a practical tool for data-driven public health management, with the potential to reduce paediatric morbidity and optimise the efficiency of health care services.

Although associations between environmental pollutants and health outcomes were observed in this study, it is important to emphasise that the ecological and cross-sectional design adopted limits causal inference. The relationships identified should therefore be interpreted as associations rather than direct causal links. Furthermore, potential confounding factors – such as pre-existing comorbidities and other urban environmental characteristics – may influence independently of climate condition and pollutant exposure. In addition, while this study discusses the potential integration of AI and big data tools into health care surveillance, it also acknowledges broader systemic barriers, including disparities in health care infrastructure, regulatory and policy limitations, and socioeconomic inequalities that may affect the implementation and effectiveness of such technological approaches. Addressing these systemic challenges is essential for ensuring that digital innovations effectively contribute to reducing health disparities and improving public health outcomes.

Our research provides robust evidence on how climatic and urban factors affect the health of vulnerable populations – particularly children and adolescents residing in large urban centres in South America. Using validated methodologies, we developed a platform capable of predicting hospital visits and admissions in a paediatric hospital in São Paulo, the most populous city in South America. Throughout 2024, São Paulo experienced some of the worst pollution levels globally, including multiple heatwaves in a single year. This situation highlights the urgent need to integrate environmental and public health policies to mitigate the impacts of climate change and urbanisation on vulnerable groups.

One potential limitation of this study concerns the exclusion of cases with low geolocation confidence scores during the data preprocessing phase. Only patient records with address geocoding at levels 8–10 on the spatial accuracy scale were retained for analysis. While this filtering step reduced the overall sample size, it was essential to ensure reliable linkage between residential location and environmental exposure data.

Importantly, this exclusion does not introduce bias in terms of representativeness, as the study does not aim to characterise the entire paediatric population of the São Paulo Metropolitan Area. Rather, the central focus is to enable an innovative approach for estimating individualised exposure levels to air pollution and weather conditions based on precise residential location and date of hospital visit. By prioritising geospatial accuracy over statistical representativeness, the study strengthens its capacity to assess associations between environmental risk factors and paediatric respiratory health outcomes.

Beyond the statistical performance of the predictive models, the results of this study offer important clinical, environmental implications and reinforces existing evidence on the vulnerability of children to environmental exposures in urban settings. Clinically, this supports the need for heightened surveillance and preventive strategies during periods of poor air quality, especially for patients with preexisting respiratory conditions. Environmentally, the ability to detect and quantify exposure at the individual level using high-accuracy geolocation strengthens the argument for integrating spatial data into paediatric health surveillance systems. This approach may inform targeted public health interventions and urban policy planning by identifying high-risk areas within the city. By linking environmental risk with clinical outcomes at a granular level, our findings underscore the importance of cross-sectoral strategies that combine health data with environmental monitoring to reduce preventable paediatric morbidity.

The platform we developed not only enhances the accuracy of health outcome forecasts but also serves as a strategic decision-support tool for public health authorities. It enables timely planning and intervention, improving the capacity to respond to environmental health threats. Our findings emphasise the value of interdisciplinary approaches in addressing the complex challenges posed by urban environmental dynamics in the context of a changing global climate.

### Limitations

The primary limitation of our study is the absence of components for analysing unstructured data from electronic medical records. These fields often contain narrative text that documents the progression of a patient’s health condition and includes valuable clinical information.

As a next step – already under way – we aim to implement deep learning algorithms capable of processing and analysing unstructured data from medical records. The goal is to incorporate this information into our platform in order to enhance the scope and depth of our analyses.

## CONCLUSIONS

We have developed a platform capable of integrating clinical databases and external data within a Big Data framework designed to process and analyse information using AI techniques applied to paediatric health.

We utilised pollution, climatic, and clinical variables to forecast emergency department visits and hospital admissions for respiratory diseases at Sabará Children’s Hospital – one of the largest paediatric hospitals in Brazil, located in the city of São Paulo. Our algorithms are able to determine whether a patient, upon arrival at the emergency department, will receive a medical consultation and be discharged or require hospital admissions. Additionally, we developed predictive models to estimate daily hospital visit and admission flows.

The final XGBoost model achieved high sensitivity, which is essential in clinical applications where early identification of paediatric patients at risk of respiratory events is critical. Minimising false negatives supports timely interventions and strengthens the potential for integrating predictive tools into real-time health monitoring systems. In contexts where environmental exposures such as air pollution can rapidly exacerbate respiratory conditions, this predictive capacity may serve as a valuable component in paediatric care strategies and public health planning. These findings highlight the promise of combining clinical and environmental data to guide early responses and mitigate preventable outcomes in vulnerable populations.

In this study, we explore how AI techniques and the application of Big Data can contribute to health research, particularly within paediatric health, as children are considered a vulnerable population. We also examine how pollution and climatic conditions can impact children's health in major urban centres across Latin America.

Our study contributes to the growing body of research on climate-related health impacts on vulnerable populations by offering insights from Brazil – a country severely affected by climate extremes and whose biodiversity and territorial scale play a key role in global climate change mitigation efforts. These findings can help support health care practices and public health strategies, while also providing valuable insights for researchers and policymakers.

This study is part of an initiative aimed at addressing the intersection between environmental issues and child health. Utilising data from Sabará Children’s Hospital, a leading paediatric hospital, combined with the expertise of the Instituto de Pesquisa e Ensino em Saúde Infantil (PENSI Institute) and financial support from the National Council for Technological and Scientific Development and the José Luiz Egydio Setúbal Foundation, this project exemplifies how research efforts can confront and mitigate the current and future challenges posed by climate change to this vulnerable population.

Through partnerships with the Climate and Sustainability Chair and the Child Health Research Group, both affiliated with the Institute of Advanced Studies at the University of São Paulo, we aim to expand this research across additional fields of study and broader territorial scales, including local, municipal, state, and national levels.
